# Repeated scoring with the adult appendicitis score improves the sensitivity and the specificity of appendicitis diagnosis in patients with early equivocal signs of appendicitis: a secondary analysis

**DOI:** 10.1007/s00068-026-03326-x

**Published:** 2026-09-03

**Authors:** Kirsi S. Lastunen, Ari K. Leppäniemi, Panu J. Mentula

**Affiliations:** 1 Helsinki University Hospital, Department of Gastrointestinal Surgery, and the University of Helsinki, Helsinki, Finland; 2 The University of Helsinki, Helsinki, Finland

**Keywords:** Appendicitis, General surgery, Acute care surgery, Scoring

## Abstract

**Purpose:**

The utilization of computed tomography in the early stage of acute appendicitis may result in overdiagnosis and unnecessarily expose patients to ionising radiation. The Adult Appendicitis Score (AAS) can be used to select patients for imaging. Observation and re-scoring in the DIAMOND trial reduced the need for imaging. Now, we wanted to determine if the change in AAS (∆AAS) can serve as a diagnostic tool to select patients for imaging even more precisely.

**Methods:**

Eighty-eight patients with early equivocal appendicitis participated in the observation arm of the DIAMOND trial. The data for these patients were reanalysed, and ∆AAS during the observation was calculated. The baseline AAS, final AAS, and the change in C-reactive protein (∆CRP) were selected as reference standards.

**Results:**

Eighty-three patients with complete data were included in the analysis. The AUROC (Area Under the Receiver Operating Characteristic) values are as follows: ∆AAS, 0.932 (95% CI 0.868–0.996); baseline AAS, 0.629 (95% CI 0.498–0.760); final AAS, 0.936 (95% CI 0.886–0.987); and ∆CRP, 0.796 (95% CI 0.696–0.897). Using receiver operating characteristic curves, we established the thresholds for low (AAS ≤ −2), intermediate (AAS −1 to 0), and high (AAS ≥ 1) probability of appendicitis. The negative predictive value for the low-probability group and the positive predictive value for the high-probability group concerning acute appendicitis were 97% and 94%, respectively.

**Conclusion:**

Patients with equivocal signs of appendicitis may benefit from short observation and the calculation of ∆AAS to reduce overdiagnosis and exposure to excessive imaging.

**Registration:**

The DIAMOND trial was officially registered on ClinicalTrials.gov (NCT02742402) on April 13, 2016.

## Introduction

Acute appendicitis constitutes one of the most common abdominal emergencies globally, and the number of patients suspected of having acute appendicitis is consequently substantial. While clear signs of appendicitis are usually easily recognised, the equivocal symptoms often prove the most problematic. Historically, the diagnosis of appendicitis relied solely on patient history and clinical examination, with observation being a common practice in ambiguous cases. Currently, in addition to patient history, clinical examination, and inflammatory markers, abdominal imaging is routinely employed. In 2010, Raja et al. [1] concluded that the routine use of computed tomography (CT) in patients suspected of having appendicitis reduced the rate of negative appendectomy from 23.0% to 1.7%. However, for the known adverse effects [2] of ionising radiation, often limited availability of imaging services, and the risk of overdiagnosing appendicitis [3, 4], the extensive use of CT cannot be recommended. Observation should remain an option for imaging when managing these patients. Observation and repeated evaluation of inflammatory markers and clinical signs have been little studied. An observational study [5] of 420 patients, in which patients were re-evaluated after a median of 6 hours, showed that the inflammatory response remained high or increased during the observation period in patients with acute appendicitis and decreased in patients without appendicitis.

Several scoring systems [6] have been introduced to improve the diagnostics of acute appendicitis, mainly to avoid overtreatment. These models use patient history, symptoms, clinical signs, and laboratory results to assign scores that categorise patients into different risk groups. This risk assessment guides the clinicians in deciding whether a patient would benefit from abdominal imaging or if the treatment plan can be made without it. The Adult Appendicitis Score (AAS) classifies patients into low-, intermediate-, and high-risk groups for acute appendicitis [7]. Patients in the intermediate-risk group usually require imaging studies to confirm the diagnosis, whereas high-score patients can be scheduled for surgery without further investigation.

We have shown in the DIAMOND trial [8] that observing patients with intermediate-risk AAS for six to eight hours is safe and effective. At the beginning of the trial, patients were randomly assigned into two groups: imaging and observation. The observation protocol reduced the need for both imaging and surgery for acute appendicitis. Observation revealed that 15 per cent of patients had spontaneously resolving appendicitis requiring no treatment. Also, by using observation protocol, 55 per cent of patients could be managed without abdominal imaging. The observation protocol did not increase the frequency of complicated appendicitis or negative appendectomies compared with the imaging group.

To our knowledge, the diagnostic performance of AAS change over time has yet to be studied. In this study, our objective was to explore this change in AAS during observation and whether this could be a valuable tool for managing patients with early equivocal appendicitis and further reducing the need for diagnostic imaging.

## Methods

The patients were recruited to the DIAMOND trial from May 3, 2016, to March 9, 2020. The prospective trial was initially registered on ClinicalTrials.gov (NCT02742402) and approved by the institutional review board and the Helsinki University Hospital ethics committee (reference number 27/13/03/02/2016). No additional approval was required for this secondary analysis. The updated STARD statement [9] was adhered to in the present study.

Before inclusion in the study, AAS was calculated for the patients. AAS ranges from 0 to 23, where 0 to 10 denotes low risk, 11 to 15 intermediate risk, and 16 to 23 high risk. The inclusion criteria were adults (18 years or older) suspected of acute appendicitis in the intermediate-risk AAS group with symptom duration of less than 24 h and C-reactive protein (CRP) level below 100 mg/l. The exclusion criteria were pregnancy, antibiotic use within the previous 24 hours, suspicion of some other illness requiring immediate intervention, prior participation in this study, and absence of written consent. The details concerning the recruitment are described in the original article [8].

The non-consecutive participants were randomly allocated in a 1:1 ratio into two arms: imaging or observation. The present study is a secondary analysis of patients in the observation arm. The clinical evaluation and laboratory testing were repeated in the observation arm after 6 to 8 hours, and the AAS was recalculated using the new variables. Patients with declining scores were discharged from the ER if no other illness was suspected. A score that did not decrease and remained below 16 led to abdominal imaging. AAS 16 or higher indicated a high risk of acute appendicitis, and an emergency appendectomy was scheduled without imaging. The diagnosis was confirmed with a histopathological analysis of all removed appendices. Complicated appendicitis is defined as perforated appendicitis or an abscess.

**Fig. 1:**
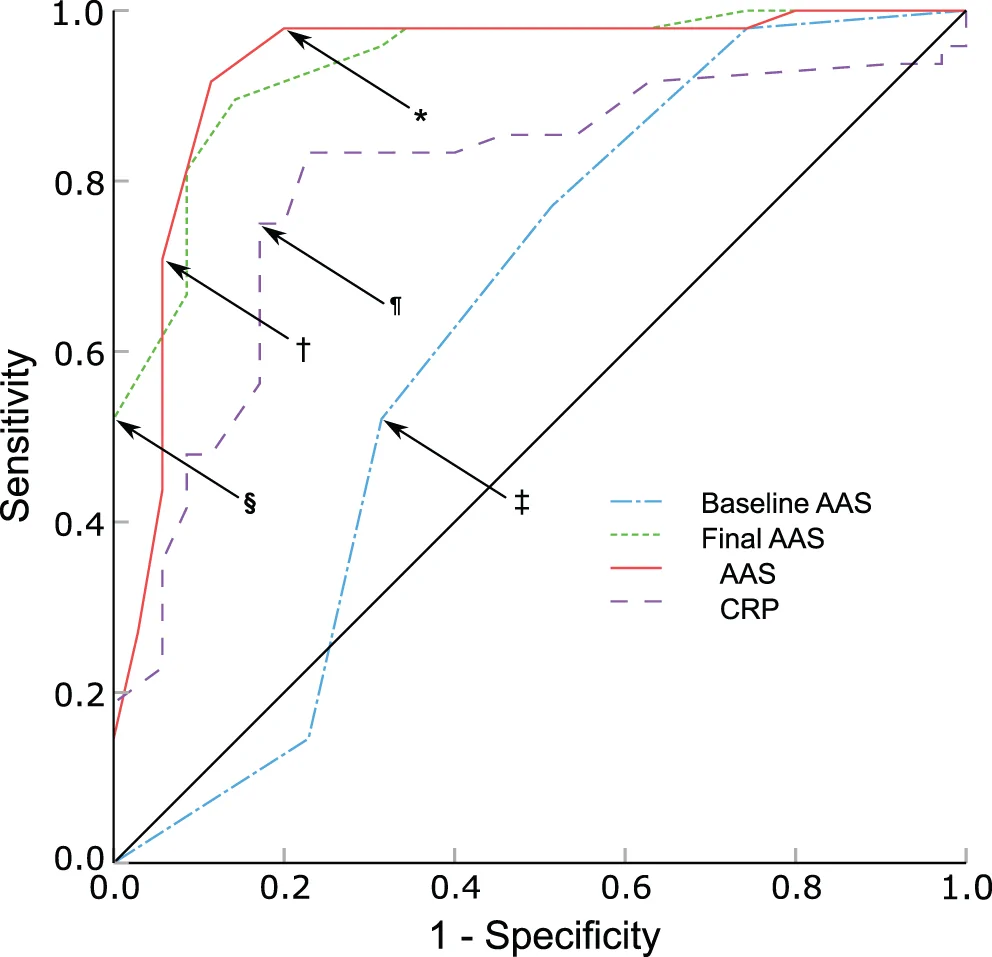
The ROC curves of the ∆AAS and the reference standards. The cut-off values are indicated in the figure. Cut-off values: *∆AAS ≥ −1, †∆AAS ≥ 1, ‡baseline AAS ≥ 14, §final AAS ≥ 16, and ¶∆CRP ≥ 9. AAS = Adult appendicitis Score, ∆AAS = Adult appendicitis Score change, ∆CRP = C-reactive protein level change

Our data recorded the AAS at the beginning (baseline AAS) and after the observation (final AAS). ∆AAS (AAS change, i.e., final AAS minus baseline AAS) is the index test. The baseline AAS, final AAS, and ∆CRP (CRP change, i.e., final CRP minus baseline CRP) were selected as reference standards. AAS is routinely used when managing patients with suspected appendicitis in our hospital. ∆CRP expresses the change in the inflammatory response during the observation period. Inkscape 1.4 was used to create graphic in Fig. 1.

## Statistical analysis

Sensitivity, specificity, positive and negative likelihood ratios (+LR and -LR), and diagnostic odds ratio (DOR) at different cut-off values of ∆AAS were calculated. A receiver operating characteristic (ROC) curve was formed using the variables of interest. The area under the receiver operating characteristic (AUROC) curve was calculated. The optimal cut-off points for ∆AAS were defined according to the LR-, DOR, sensitivity and specificity values, and the ROC curve. These cut-off points categorised patients into three risk groups after observation. The cut-off points for the reference standards were also determined according to their respective ROC curves. McNemar’s test was used to test whether the need for imaging could be reduced using the new risk assessment compared with the number of patients imaged during the trial. The statistical analysis was accomplished using SPSS® version 29 (IBM, Armonk, NY, USA).

## Results

Eighty-eight patients in the observation group received the allocated intervention in the DIAMOND trial. In the present study, four patients were excluded due to protocol violations because their final AAS was not recorded. Also, one patient with radiologically diagnosed appendicitis treated nonoperatively was excluded, as we did not have the histopathological confirmation of acute appendicitis. In the end, 83 patients were available for analysis in this study.

The median age of the patients was 29 (IQR 25–38), and 43 (52%) were female. Table 1 describes the demographics and clinical characteristics of the patients.

**Table 1:** Demographics and clinical characteristics

Age, years^*^	29 (25–38)
Female	43 (52)
Duration of symptoms, hours^†^	12.7 (5.6)
Baseline CRP, mg/L^*^	6.0 (3.0–14.0)
Baseline WBC count, E9/L^*^	12.3 (10.0–15.0)
Baseline AAS^*^	13 (12–14)

Values in parentheses are percentages unless indicated otherwise; values are *median (IQR) and †mean (s.d.). CRP = C-reactive protein, WBC = white blood cell, AAS = Adult Appendicitis Score

Twenty-five (30%) patients had a final AAS of 16 or higher, and the decision to operate was made without imaging. All these patients had appendicitis; two of them had complicated appendicitis. Thirty-one (37%) patients underwent abdominal imaging after the re-scoring, resulting in 24 surgeries for acute appendicitis, one of which was a negative appendectomy. Twenty-seven patients (33%) were discharged without imaging. None of these patients returned due to appendicitis within the first 30 days. In the end, forty-eight (58%) patients were diagnosed with histologically confirmed appendicitis.

The receiver operating characteristic curves (Fig. 1) and the AUROC values (Table 2) show that both the ∆AAS and final AAS perform better in diagnosing acute appendicitis (ROC area values 0.932 (95%CI 0.868–0.996) and 0.936 (95%CI 0.886–0.987)) compared to baseline AAS and ∆CRP (ROC area values 0.629 (95%CI 0.498–0.760) and 0.796 (95%CI 0.696–0.897)).

**Table 2:** The values of AUROC for the ∆AAS and the reference standards

	AUROC	SE	p
Baseline AAS	0.629 (0.498–0.760)	0.067	0.046
Final AAS	0.936 (0.886–0.987)	0.026	<0.001
∆AAS	0.932 (0.868–0.996)	0.033	<0.001
∆CRP	0.796 (0.696–0.897)	0.051	<0.001

95% confidence interval in parentheses. AUROC = area under the receiver operating characteristics, SE = standard error, AAS = Adult Appendicitis Score, *Δ *AAS = change in Adult Appendicitis Score, *Δ *CRP = change in C-reactive protein. *p*-values were determined with the Mann-Whitney U-test

To identify the low, intermediate, and high probability of appendicitis after observation, two cut-off values for ∆AAS were determined using the ROC curves: ∆AAS ≥ −1 and ∆AAS ≥ 1. For comparison, the reference standards’ cut-off values were also determined: baseline AAS ≥ 14, final AAS ≥ 16, and ∆CRP ≥ 9, Fig. 1. The sensitivity, specificity, likelihood ratios, and diagnostic odds ratios of acute appendicitis were calculated for specific ranges of baseline AAS, final AAS, ∆AAS, and ∆CRP, as shown in Table 3. These cut-off values are indicated in Fig. 1.

**Table 3:** Defining the cut-off values for ∆AAS and the reference standards

	∆AAS ≥ −1	∆AAS ≥ 1	Baseline AAS ≥ 14	Final AAS ≥ 16	∆CRP ≥ 9
Sensitivity	97.9% (89.1–99.6%)	70.8% (56.8–81.8%)	52.1% (38.3–65.5%)	52.1% (38.3–65.5%)	75.0% (61.2–85.1%)
Specificity	80% (64.1–90.0%)	94.3% (81.4–98.4%)	68.6% (52.0–81.4%)	100.0% (90.1–100.0%)	80.0% (64.1–90.0%)
+LR	4.90 (2.52–9.51)	12.40 (3.19–48.20)	1.66 (0.95–2.90)	infinity	3.75 (1.90–7.42)
-LR	0.03 (0.004–0.18)	0.31 (0.20–0.48)	0.70 (0.51–0.96)	0.48 (0.36–0.64)	0.31 (0.19–0.52)
DOR	188.00 (21.97–1608.95)	40.07 (8.45–190.14)	2.37 (0.95–5.90)	infinity	12.00 (4.18–34.46)

95% confidence interval in parentheses. ∆AAS = change in Adult Appendicitis Score, AAS = Adult Appendicitis Score, ∆CRP = change in C-reactive protein, +LR = positive likelihood ratio, -LR = negative likelihood ratio, DOR = diagnostic Odd’s ratio

**Fig. 2:**
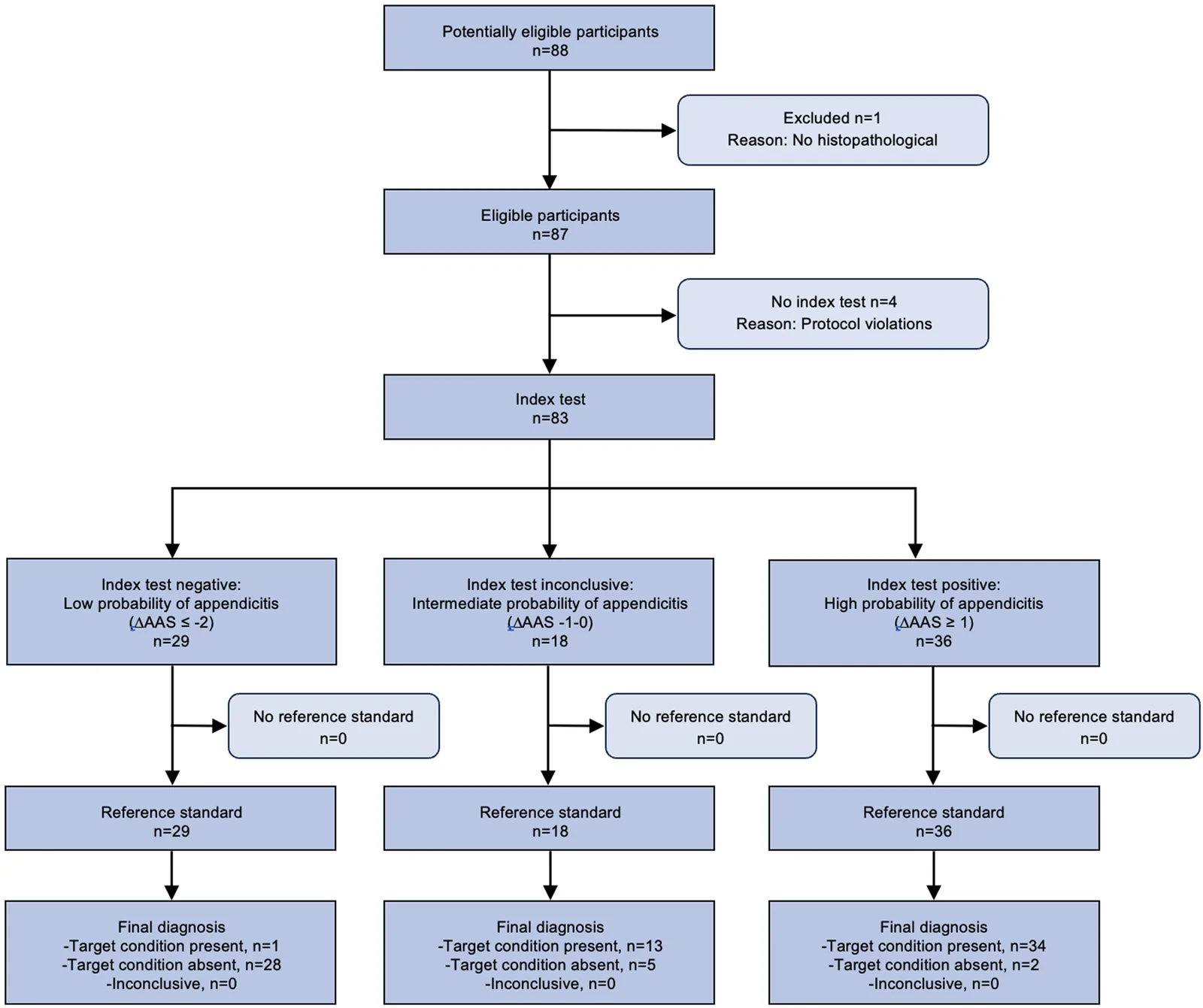
Flow of participants. ∆AAS = Adult appendicitis Score change

Based on the chosen cut-off values of ∆AAS, patients can now be classified into three groups corresponding to the probability of appendicitis: low (∆AAS ≤ −2), intermediate (∆AAS −1–0), and high (∆AAS ≥ 1) probability. The proportions of acute appendicitis were then calculated in these three groups (Table 4). There were 29 (35%) patients in the low-probability group, and only 1 (3%) had acute appendicitis. Eighteen (22%) patients fell into the intermediate probability group, and 13 (72%) of them had acute appendicitis. There were 36 (43%) patients in the high probability group, and 34 (94%) of them had acute appendicitis. The negative predictive value of the low (∆AAS ≤ −2) probability of acute appendicitis is 97 per cent, and the positive predictive value of the high (∆AAS ≥ 1) probability of acute appendicitis is 94 per cent. The flow of patients into these groups is presented in Fig. 2.

**Table 4:** Three groups: low, intermediate, and high probability of acute appendicitis

	Low: ∆AAS ≤ −2	Intermediate: ∆AAS −1 - 0	High: ∆AAS ≥ 1
N	29 (35)	18 (22)	36 (43)
Acute appendicitis diagnosis	1 (3)	13 (72)	34 (94)

Values in parentheses are percentages. ∆AAS = change in Adult Appendicitis Score

Thus, utilising these groups based on ∆AAS, only the 18 (22%) patients in the intermediate probability group would have benefited from imaging studies. Compared with the 31 (37%) patients actually imaged during the trial according to the protocol, this is a significantly lower number of patients, *p* < 0.001.

## Discussion

During observation of patients with equivocal signs of appendicitis, the measurable inflammatory response and clinical findings evolve, causing changes in AAS. Repeated scoring allows the change in AAS (∆AAS) to be used as a diagnostic tool. This study showed that the ∆AAS during the observation period has significantly better diagnostic accuracy and overall sensitivity than baseline AAS. Patients with appendicitis showed increasing CRP levels, but the diagnostic accuracy of the CRP change was markedly poorer than that of the ∆AAS. In line with these results, an earlier study [5] demonstrated that CRP change had independent diagnostic value in addition to the final CRP value after observation of patients with equivocal signs of appendicitis.

In the DIAMOND trial [8], we showed that the need for diagnostic imaging was considerably lower when the AAS-based observation protocol was utilised compared with routine imaging. Observation and re-scoring with AAS can further reduce the need for imaging if the ∆AAS is used instead of a predefined cut-off score of 16 or more. ∆AAS can then be used to categorise patients into three probability groups. The risks of missing appendicitis in the low-probability group or ending up with negative appendectomy in the high-probability group are small. Thus, because patients in the high- and low-probability groups would not benefit from imaging, the imaging rate could be reduced considerably. This classification can speed up decision-making after observation, free up imaging resources in the emergency department for other patients, and reduce the cancer risk [2] associated with ionising radiation from CT.

The increased use of CT has led to excess detection of uncomplicated appendicitis, as CT cannot differentiate self-resolving appendicitis from perforating appendicitis. This has resulted in overdiagnosis of appendicitis [3, 4]. As previously concluded in the DIAMOND trial [8], observation and avoidance of early imaging can help distinguish patients with spontaneously resolving appendicitis. Most patients could be managed without imaging, and considerably fewer patients required treatment for appendicitis. This new classification can further reduce overtreatment by reducing excess imaging. As shown in the DIAMOND-trial and in a more recent PERFECT-trial [10], observation or longer delay to surgery does not increase the perforation rate or postoperative complications, thus making short (up to 8 hours) observation a safe alternative to early imaging.

According to the prospective validation study [11], the AAS intermediate-risk group comprises 39 per cent of all patients suspected of acute appendicitis. Only this group of patients is generally recommended to be imaged: ultrasound first and CT if appendicitis is not detected. Considering observation with the new classification based on ∆AAS, the overall proportion of patients suspected of appendicitis needing imaging could be reduced substantially.

As this study shows, there are many ways to improve observation protocols further. Utilisation of ∆AAS may be one way. Selecting suitable patients for observation is another critical area that requires further studies. Using scoring systems that differentiate between patients with uncomplicated and those with complicated appendicitis may also be helpful. Unfortunately, such a scoring system that does not use imaging studies for differentiation has not been published to date.

This study has limitations. Only patients with less than 24 hours of symptom duration and CRP levels below 100 mg/l were included in the study. Also, the number of patients in the observation arm is small. These results should be confirmed in a more extensive, preferably prospective, study with less stringent inclusion criteria.

## Conclusion

Re-scoring with AAS after six to eight hours of observation is a worthy and feasible alternative to imaging in patients within the AAS intermediate risk group of appendicitis. The change in AAS can be utilised as a diagnostic tool, thereby reducing the need for imaging and easing decision-making when treating patients with acute appendicitis.

## Data Availability

Data sharing is not feasible because GDPR mandates us to safeguard participants’ identities, preventing us from sharing raw data publicly.
